# Development of IgE-mediated food allergies in children with history of food protein-induced allergic proctocolitis: a series of five cases

**DOI:** 10.3389/falgy.2024.1354106

**Published:** 2024-03-28

**Authors:** Kim L. Tran, Elizabeth L. Wisner, George M. Jeha, Luke A. Wall

**Affiliations:** ^1^ School of Medicine, Louisiana State University Health Sciences Center New Orleans, New Orleans, LA, United States; ^2^ Allergy and Immunology, Children’s Hospital of New Orleans, New Orleans, LA, United States

**Keywords:** case report, food allergy, atopy, FPIAP, anaphylaxis, IgE

## Abstract

Food protein-induced allergic proctocolitis (FPIAP) is a non-IgE-mediated allergic condition that presents with hematochezia in otherwise healthy infants. It is most commonly induced by cow's milk protein via breast milk or formula. The prognosis for FPIAP is generally considered favorable with most infants achieving symptomatic resolution after diet modification. Most infants go on to tolerate the offending foods by 1–3 years of age. Over 8 years at our institution, five patients were identified and noted to have FPIAP to cow's milk during infancy with subsequent development of IgE-mediated allergic reaction to cow's milk and other foods. All five cases developed other atopic disorders (atopic dermatitis in four cases). IgE-mediated cow's milk allergy has persisted beyond the preschool years in at least two patients (currently 8 and 16 years old). For three of the patients, the IgE-mediated reaction to cow's milk was severe with development of anaphylaxis or angioedema. In addition, three patients experienced anaphylaxis or angioedema to allergens other than milk. While FPIAP is a non-IgE-mediated process traditionally thought not to progress past the first year of life, some infants with FPIAP develop severe, persistent IgE-mediated cow's milk allergy. To our knowledge, this is the first detailed clinical description of such patients.

## Introduction

Food protein-induced allergic proctocolitis (FPIAP) is a non-IgE-mediated response to food that leads to colonic inflammation and hematochezia. It presents most commonly within the first two months of life but has been reported in infants as young as 1 week and up to 6 months of age (1). FPIAP is a fairly common condition in breastfeeding infants with a prevalence ranging from 0.16% to 17% in healthy infants and accounting for 64% of all infants presenting with bloody stools (1, 2). The most common offending food in FPIAP is cow's milk (3). Several other foods have been implicated in FPIAP including egg, wheat, and soy, among others. Overall, the prognosis for FPIAP is good with total tolerance of offending foods occurring by 1–3 years of age without recurrence of symptoms in most infants (1).

The diagnostic criteria for FPIAP include the presence of bloody stools that resolve when the offending food is removed from the diet and recurrence of symptoms upon reintroduction. In most cases, the symptoms resolve within 48–72 h after modification of the infant's diet but can take up to 1–2 weeks in breastfed infants (1). In clinical practice, when resolution of bloody stools is rapid and apparent, immediate reintroduction of the offending food to demonstrate recurrence is rarely performed. Other potential causes of rectal bleeding including infection, anal fissure, intussusception, or inflammatory bowel disease (IBD) (1) should be excluded. Although usually not required, endoscopy with biopsy can be performed to confirm the diagnosis (1). Expected endoscopic findings include focal erythema with lymphoid nodular hyperplasia (4). The signature histologic findings include marked eosinophils and degranulation in the rectosigmoid colon in close proximity to lymphoid nodules (4).

Despite its prevalence, the pathogenesis of FPIAP is not well understood. Several theories have been proposed that center around increased levels of pro-inflammatory cytokine tumor necrosis factor-alpha (TNF-α) (5) and anti-inflammatory cytokine transforming growth factor-beta (TGF-β) (6, 7). TNF-α increases the permeability of the gut epithelium, which may lead to increased inflammation and immune reaction to otherwise benign food proteins. TGF-β, produced by regulatory T cells (T_reg_), normally dampens immune response and is responsible for immune tolerance. Decreased levels of TGF-β in patients with FPIAP may consequently lead to reduced tolerance to food proteins (5, 6). T helper 2 (T_H_2) cells via production of interleukin-4 (IL-4) and subsequent IgE are responsible for IgE-mediated food allergies but have also been implicated in non-IgE-mediated food allergies by production of cytokines IL-3, IL-5, and IL-13 (6, 7). The exact role of T cells in the pathogenesis of FPIAP is poorly understood.

The gut-associated lymphoid tissue (GALT) allows the gut to be poised both for robust immune response to pathogens and critical tolerance to non-harmful foreign antigens including food peptides and normal microbiota. In recent years, the innate lymphoid cells (ILCs) have gained much attention and play a significant role in the gut where they function as regulators of tissue homeostasis, inflammation, and early innate response to infection (8). ILC2s lack antigen-specific receptors but share functional similarities with the T_H_2 cell population and produce the T_H_2-associated cytokines IL-4, IL-5, and IL-13 (8, 9). The proliferation and activation of ILC2s is supported by the predominantly epithelial cell-derived cytokine “alarmins,” IL-25, IL-33, and thymic stromal lymphopoietin (TSLP). These cytokines are released by epithelial cells in response to inflammation and are completely independent of an adaptive immune response (8). It is therefore plausible that ILC2s may play a role in FPIAP, especially considering the immaturity of the adaptive immune system in early infancy. Cytokines produced by ILC2s may subsequently activate mast cells and impede allergen-specific T_regs_ (9) while driving eosinophilic inflammation, inducing T_H_2 differentiation, and ultimately B cell class switching to IgE (10, 11). Therefore, while their precise role in FPIAP requires further investigation, ILC2s appear to be at least one plausible mechanism bridging an initial mucosal-involved non-IgE-mediated reaction (FPIAP) with subsequent IgE sensitization.

There is a mounting body of evidence that the gut microbiota is impacted directly by diet, inflammation, and environment, and the microbiota subsequently impacts disease state (12). Recent data from the Gastrointestinal Microbiome and Allergic Proctocolitis study demonstrated key differences in the microbiome of infants with FPIAP, specifically a higher abundance of a genus of Enterobacteriaceae and a lower abundance of a family of Clostridiales during the symptomatic period, with some differences noted prior to symptom onset (13). The authors concluded that complex cross talk between the intestinal microbiome, food antigens, intestinal inflammation, and the innate immune system early in life likely contributes to the mechanisms responsible for either healthy tolerance or food allergy development (13).

Significant differences exist between the natural history of IgE-mediated vs. non-IgE-mediated cow's milk allergy. While there is heterogeneity in the available data regarding the timing of the resolution of IgE-mediated cow's milk allergy in children, an article compiling nine studies on the topic suggests that IgE-mediated cow's milk allergy resolves by age 5 years in 50% of the cases and by age 16 years in 80% of the cases (14). As for the severity of IgE-mediated cow's milk allergy, only 3% of the 139 children in a study by Santos et al. presented with anaphylaxis with the majority of reactions being cutaneous (81%) or gastrointestinal symptoms (55%) (15). Therefore, persistent IgE-mediated cow's milk allergy with severe symptoms is not commonly seen.

For non-IgE-mediated cow's milk allergy such as FPIAP, the results are even more striking. In one study looking at a large European cohort, all children with non-IgE-mediated cow's milk allergy tolerated cow's milk after 1 year of diagnosis compared with 57% of the children with IgE-mediated cow's milk allergy (16). Most symptoms associated with non-IgE-mediated food allergy are gastrointestinal in nature as seen by Meyer et al. and range from vomiting, diarrhea, abdominal pain, to rectal bleeding (17). Although the current state of clinical practice separates FPIAP from IgE-mediated food allergy as two unrelated conditions, at least one study in recent years has documented an increased risk for subsequent development of IgE-mediated food allergy in children who experienced FPIAP, although the study was not set up to elucidate clinical details of specific cases (18). This report is unique in that it describes the characteristics of five infants with a history of FPIAP who subsequently developed severe and persistent IgE-mediated allergy to cow's milk.

## Materials and methods

The institutional review board (IRB) approval was obtained through the Louisiana State University Health Sciences Center, and the chart review was conducted in the Children's Hospital New Orleans Allergy and Immunology clinics. Data were de-identified and compiled in Microsoft Excel spreadsheet for analysis.

## Results

### Case 1

**Figure 1:**
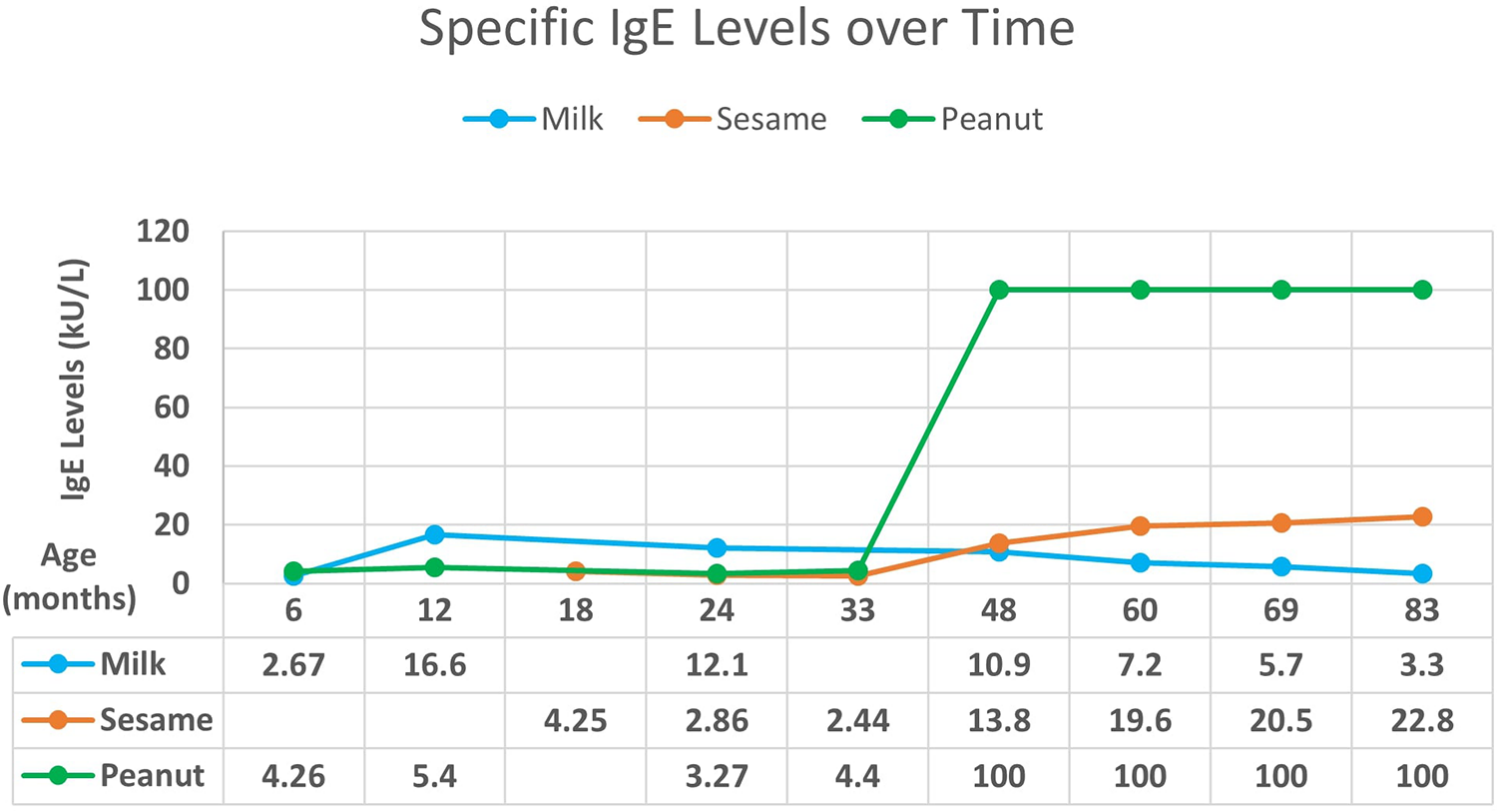
Case 1: specific IgE levels against age.

A male breastfed infant with a history of atopic dermatitis and failure to thrive presented with hematochezia in the first 3 months of life, exact age unknown. The hematochezia resolved with elimination of cow's milk from the maternal diet. At 5 months of age, he developed urticaria and angioedema with ingestion of cow's milk-based formula. Allergy testing at 6 months of age revealed increased specific IgE to milk and peanuts (Figure 1). Strict avoidance of milk and peanuts was advised. At 2 years of age, he passed an oral challenge to extensively heated (“baked”) milk and was instructed to continue regular ingestion of this food.

At 18 months of age, the patient experienced acute urticaria and angioedema after ingestion of hummus, which was attributed to sesame. Specific IgE to sesame was positive. Over time, food allergy testing showed decreasing specific IgE levels to milk with increasing levels to sesame and peanuts (Figure 1) with levels being exceedingly high for peanuts. Despite decreasing milk IgE and tolerance to extensively heated milk, the patient continues to experience clinical reactivity including oral pruritus with small ingestions of unheated milk protein. Exposure to peanuts without direct ingestion induces a cough. The patient is currently 8 years old and continues avoidance of unheated milk, peanuts, and sesame.

### Case 2

A 2-week-old breastfed male, with subsequent development of atopic dermatitis, reflux, and failure to thrive, presented with hematochezia. The hematochezia resolved with elimination of cow's milk from the maternal diet.

**Figure 2:**
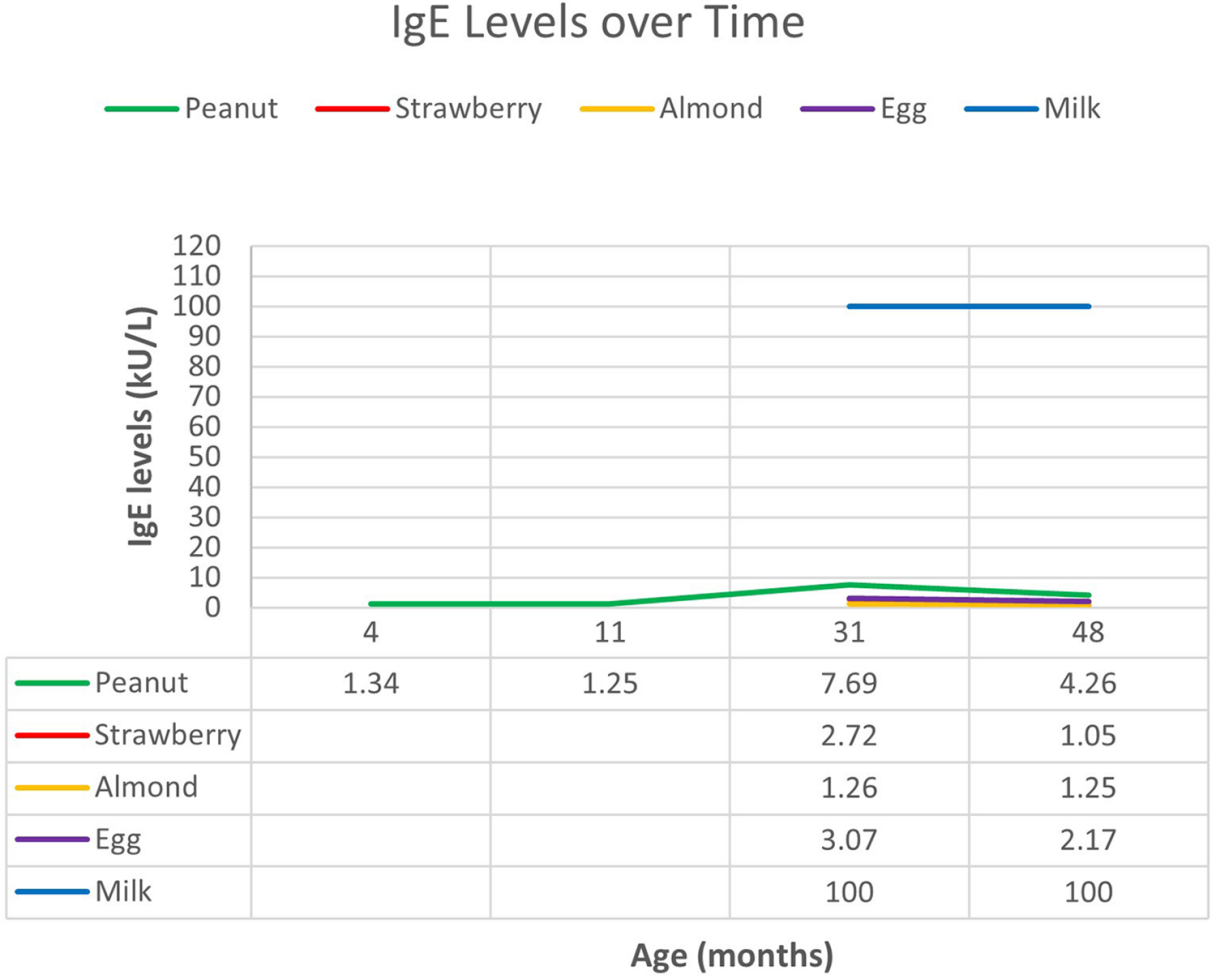
Case 2: specific IgE levels against age.

At 9 months of age, the patient presented to the allergy clinic for evaluation of atopic dermatitis. Allergy testing performed by an external provider was positive to peanuts. Repeat testing demonstrated elevated IgE to peanuts and negative IgE to cow's milk (Figure 2).

The patient returned at 1 year of age after developing anaphylaxis with ingestion of cow's milk-based yogurt. Heated forms of milk such as baked goods and waffles were tolerated. Skin prick testing to milk was positive (7 mm × 3 mm of induration). Continued regular ingestion of baked milk was advised.

Subsequent skin prick testing and serum specific IgE testing was positive for eggs, peanuts, and tree nuts and was exceedingly high for milk. He also later developed anaphylaxis to strawberry, which was confirmed with specific IgE testing. Spontaneous episodes of urticaria were reported at 2 years of age, occurring every 1–2 weeks without a specific trigger. These episodes persisted alongside spontaneous episodes of vomiting, and he was later diagnosed with cyclic vomiting at age 3. Importantly, baseline serum tryptase level was normal at multiple timepoints. Raw cow's milk products have not been reintroduced due to persistence of exceedingly high specific IgE to milk, prior history of anaphylaxis, and recent urticaria after eating powdered-cheese-coated crackers. Although he is currently 8 years of age, he has been lost to follow-up. His last testing was performed at 4 years of age.

### Case 3

A 10-month-old male presented to clinic for evaluation of atopic dermatitis and hematochezia while consuming cow's milk-based formula. The hematochezia resolved with transition to an extensively hydrolyzed formula.

At 11 months of age, the patient developed urticaria after ingesting a cookie containing cow's milk. Skin prick testing to milk was positive (11 mm × 13 mm induration). Strict cow's milk avoidance was advised. The patient has not returned to clinic for follow-up but has been treated in the emergency department (ED) twice for allergic reactions to an unknown candy (age 2 years) and mixed nuts (age 3 years). He is currently 3 years of age.

### Case 4

**Figure 3:**
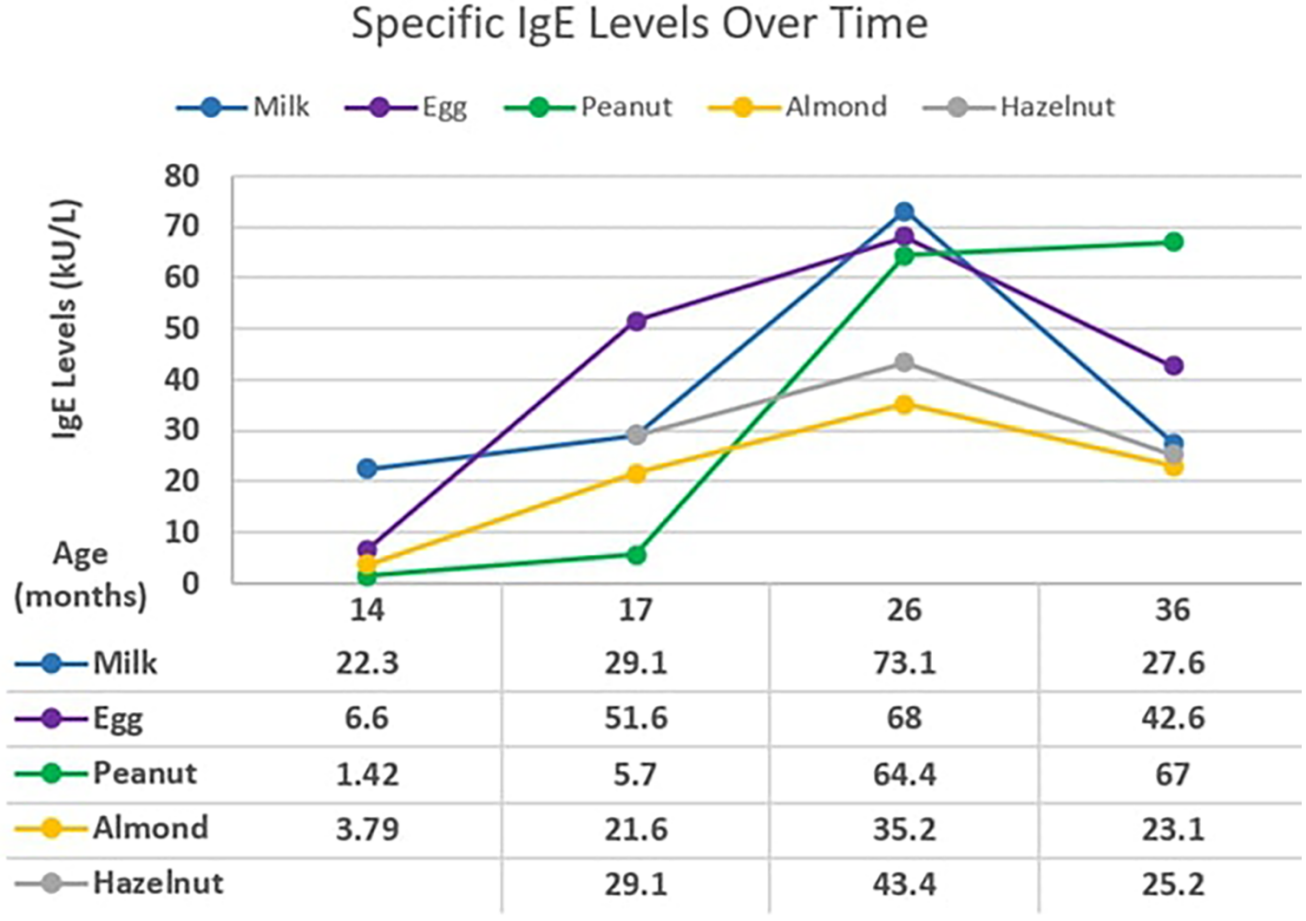
Case 4: specific IgE levels against age.

A 2-month-old male with atopic dermatitis presented with hematochezia while on cow's milk-based formula. The hematochezia resolved after changing to soy-based formula. He later developed urticaria with macaroni and cheese and cow's milk-based yogurt at 8 months and 9 months of age, respectively. The initial testing revealed specific IgE to milk, eggs, almonds, hazelnuts, and peanuts (Figure 3). Extensively heated forms of milk and eggs were tolerated.

At 17–26 months of age, testing revealed increasing specific IgE levels to milk, eggs, almonds, peanuts, and hazelnuts. At 36 months of age, peanut IgE continued to increase while other foods have trended downward. Specific IgE levels have been exceedingly high for milk, eggs, and peanuts. Since his initial reactions to cow's milk, he has continued to tolerate baked cow's milk and now tolerates melted cheese on pizza. He is currently 3 years of age.

### Case 5

A 1-week-old male on cow's milk-based formula presented with hematochezia. The hematochezia resolved when he was transitioned to amino acid-based formula. At 1 year of age, the patient developed anaphylaxis after consuming a cheese-containing puffed snack. Interestingly, the patient developed acute onset of cough whenever his mother cooked with cow's milk. Per history, the patient first presented to an outside allergist at 2 years of age where he tested positive to milk, peanuts, beef, soy, and wheat, although these records were not available for review.

Upon presentation to our clinic at 15 years of age, he was noted to have exercise-induced asthma and allergic rhinitis. He reported clinical tolerance to beef, soy, and wheat but strictly avoided milk, peanuts, and tree nuts. Allergy testing revealed elevated specific IgE to milk, peanuts, and cashew nuts. He has strictly avoided cow's milk owing to continued symptoms of cough when milk is being cooked. He is currently 16 years of age.

## Summary of cases

In summary, these five patients with FPIAP subsequently developed IgE-mediated allergy to cow's milk and other foods. The initial IgE-mediated reaction occurred at a mean age of 9.6 months, with a range of 5–12 months of age. IgE-mediated cow's milk allergy has persisted beyond the preschool years in at least two patients (currently 8 and 16 years old). A third patient demonstrated exceedingly high IgE to milk with clinical reactivity when lost to follow-up at age 4. For three of the patients, the IgE-mediated reaction to cow's milk was severe with development of anaphylaxis or angioedema. In addition, three patients experienced anaphylaxis or angioedema to allergens other than milk. Allergies to peanuts and milk appeared to be the most prevalent. Allergen-specific IgE levels were exceedingly high, with two patients having cow's milk IgE >60 kU/L and two patients having peanut IgE >60 kU/L. All five patients are male, and all developed other atopic diseases including four with atopic dermatitis. A summary of the cases is provided in Table 1 for side-by-side comparison.

**Table 1:** Case summaries.

Case details	Resolution of bloody stools in infancy	IgE-mediated food reactions	Positive allergy testing	Atopic history
1 Sex: M Age at FPIAP: <3 months 1st IgE reaction: 5 months Current age: 8 years	Removal of milk from maternal diet while breastfeeding	Milk-based formula → urticaria, angioedema (5 months) Hummus → anaphylaxis (18 months)	Milk, peanut, sesame	Atopic dermatitis
2 Sex: M Age at FPIAP: 2 weeks 1st IgE reaction: 1 year Current age: 8 years	Removal of milk from maternal diet while breastfeeding	Cow's milk-based yogurt → anaphylaxis (12 months) Strawberry → anaphylaxis, urticaria (12 months)	Milk, peanut, egg, tree nuts, strawberry	Atopic dermatitis Family history of atopic dermatitis
3 Sex: M Age at FPIAP: 10 months 1st IgE reaction: 11 months Current age: 3 years	Switching to extensively hydrolyzed formula	Baked milk → urticaria (11 months) Mixed nuts → angioedema (3 years)	Milk (did not return for testing to nuts)	Atopic dermatitis
4 Sex: M Age at FPIAP: 2 months 1st IgE reaction: 8 months Current age: 3 years	Switching to soy-based formula	Macaroni and cheese, cow's milk-based yogurt → urticaria (8–9 months)	Milk, peanut, egg, almond, hazelnut	Atopic dermatitis Family history of atopic dermatitis, asthma, food allergy in father (unspecified)
5 Sex: M Age at FPIAP: 1 week 1st IgE reaction: 1 year Current age: 16 years	Switching to amino-acid-based formula	Cheese-flavored puff → anaphylaxis (12 months)	Milk, peanut, cashew	Exercise-induced asthma, allergic rhinitis

## Discussion

Despite its prevalence, little is known about the pathogenesis of FPIAP and its possible complications. IgE-mediated allergic reactions such as those occurring in our patients appear to be potential sequelae of FPIAP. The age at diagnosis of IgE-mediated food allergy in our report aligns with the general population: median (and interquartile range) was 12 (7–19) months for milk allergy in an observational cohort of over 200,000 children (18). However, high severity and long persistence of cow's milk allergy as seen in our patients are unexpected relative to the typical natural history of IgE-mediated cow's milk allergy (7). Although persistence does not have a strict definition in the literature, we define persistence as continual clinical reactions to certain food proteins or continually elevated specific IgE levels that extend beyond the timeframe of what is expected from its natural history. In our cases for example, a persistent IgE-mediated milk allergy would be the presence of symptoms after the ingestion of milk proteins or elevated IgE levels to milk protein in a child over the age of 1 year with a history of FPIAP, as most—if not all—children with a history of FPIAP tolerate milk proteins by age 1. All of our cases, therefore, demonstrate persistence of allergy to milk proteins. This is especially striking in Case 5 as the allergy has persisted into his teenage years. Overall, these observations suggest that there could be an underlying predisposing factor leading to both FPIAP and subsequent severe and persistent IgE-mediated food allergy in some infants.

One factor that serves as a common thread linking this limited number of cases appears to be the development of atopy, specifically atopic dermatitis. The traditional teaching of allergy centers on sequence and timing, and it is widely accepted that active inflammation of eczematous skin predisposes to sensitization to food antigens via skin exposure. In general, a link between atopic dermatitis and food allergy is well accepted as demonstrated in a systematic review conducted by Tsakok et al. (19).

However, infants develop FPIAP in the immediate neonatal period, and as demonstrated by our cohort, subsequently develop atopic dermatitis and IgE-mediated food allergy. The development of this “non-traditional allergic march” that begins with FPIAP and leads to IgE-mediated food allergy has been documented in at least one study in recent years. In the 903 healthy infants analyzed by Martin et al., 153 developed FPIAP and 56 developed IgE-mediated food allergy. Infants who had IgE-mediated food allergy were more likely to have atopic dermatitis while atopic dermatitis also occurs in many patients with FPIAP. The study observed that 11% of FPIAP patients developed IgE-mediated food allergy compared with the 5% who did not have FPIAP, although details regarding food reactions were sparse. Overall, their analysis found that the patients with FPIAP had twice the odds of developing IgE-mediated food allergy after adjusting for atopic dermatitis (20). The authors went on to propose two possible mechanisms for the development of IgE-mediated food allergy: first, the predominantly T_H_2 response implicated in non-IgE-mediated food reactions may predispose children with FPIAP to IgE-mediated food allergy. Second, elimination of a specific food from the diet—a strategy used to treat FPIAP— may increase the risk of IgE-mediated food allergy.

Regarding allergen introduction, it is known that early introduction of allergenic foods is effective in preventing the development of food allergy, especially in higher risk infants with underlying atopic dermatitis (21). The LEAP trial strongly demonstrates that early and regular ingestion of peanut protein is effective in preventing peanut allergy (22). While infants with FPIAP to cow's milk obviously cannot tolerate cow's milk in the earliest weeks of life, the concept of reintroduction at the earliest timepoint possible, in the most tolerable form for that timepoint (baked ingredient or partially hydrolyzed formula, for example) would likely be very important to mitigate the risk of developing IgE-mediated food allergy. Since there is a risk of bleeding recurrence after food rechallenge (23), FPIAP is currently managed with food reintroduction after the culprit food has been eliminated from the diet for 6 months or at 1 year of age—whichever comes first. In the aforementioned cases, the approach to early introduction of other potentially allergenic foods was highly variable owing to multiple factors: parental fear leading to unnecessary avoidance and/or empiric testing, initial testing and advice given by other providers prior to referral to the allergy clinic, and the fact that many of these children were born prior to the widespread emphasis on early introduction of foods brought about by the landmark LEAP trial (22). Research and clinical guidance regarding earliest reintroduction or tolerability of milk products in infants with history of FPIAP is lacking. The lack of any laboratory test or biomarker for the degree of active non-IgE-intolerance to the culprit food is another challenge when reintroduction is being considered. In patients with FPIAP and especially in those with atopic dermatitis, specific IgE testing to the culprit food may be considered prior to reintroduction to weigh the potential risk of an IgE-mediated reaction.

Although this report has significant limitations inherent to the fact that it is a retrospective case series and the clinical approach as well as diagnostic methods were very heterogeneous, with many of the patients initially evaluated in other allergy clinics or by primary care, important observations should be made. While observational data collected from cases cannot imply causality, there appears to be a connection between FPIAP, IgE-mediated food allergies, and atopy as demonstrated by the five cases illustrated in this article, a finding also supported by the limited available literature. We propose that the inflammation occurring in the infant colon during active FPIAP must be questioned as a potential factor leading to immunologic priming and subsequent IgE-mediated sensitization to foods. Furthermore, perhaps some infants could have a predisposing genetic or epigenetic factor leading to FPIAP, subsequent development of atopy, and IgE-mediated food allergy that is more severe and persistent than usual. While limited studies have shown an increased risk of development of IgE-mediated allergy in infants who previously experienced FPIAP, this risk is not a traditionally held view and therefore not widely recognized in clinical practice. In addition, previous reports documented only the percentage of infants who went on to develop IgE-mediated food allergy without providing extensive clinical details. To our knowledge, this is the first case series of patients with FPIAP who developed severe and persistent IgE-mediated food allergies including to allergens such as cow's milk that is otherwise expected to be outgrown in early childhood.

## Conclusions

Based on the observations documented in this report and limited published evidence, it would be worthwhile for infants presenting with FPIAP to be referred to an allergist and for culprit-food-specific IgE testing to be performed prior to reintroduction of the food. An oral food challenge may be considered for infants with low-level positive IgE testing and without history of severe IgE-mediated reaction, as many of these infants will tolerate the food at a young age (24). Considering the current knowledge gap regarding the risk for development of IgE-mediated allergy in these infants and the potential life-threatening reactions suggested by this report, there is an urgent need for additional studies, awareness, and education regarding the potential sequelae of FPIAP. More studies are needed regarding the pathogenesis, natural history, potential biomarkers, and timing and method of food reintroduction.

## Data Availability

The original contributions presented in the study are included in the article/Supplementary Material, further inquiries can be directed to the corresponding author.
